# Metformin and Vitamin D Modulate Inflammation and Autophagy during Adipose-Derived Stem Cell Differentiation

**DOI:** 10.3390/ijms22136686

**Published:** 2021-06-22

**Authors:** Sara Cruciani, Giuseppe Garroni, Renzo Pala, Maria Laura Cossu, Giorgio Carlo Ginesu, Carlo Ventura, Margherita Maioli

**Affiliations:** 1 Department of Biomedical Sciences, University of Sassari, Viale San Pietro 43/B, 07100 Sassari, Italy; sara.cruciani@outlook.com (S.C.); giugarroni21@gmail.com (G.G.); renzopala6@gmail.com (R.P.); 2 General Surgery Unit 2 “Clinica Chirurgica”, Department of Medical, Surgical and Experimental Sciences, University of Sassari, Viale San Pietro 8, 07100 Sassari, Italy; mlcossu@uniss.it (M.L.C.); ginesugc@uniss.it (G.C.G.); 3 Laboratory of Molecular Biology and Stem Cell Engineering, National Institute of Biostructures and Biosystems Eldor Lab, Innovation Accelerator, Consiglio Nazionale delle Ricerche, 40129 Bologna, Italy; ventura.vid@gmail.com; 4 Center for Developmental Biology and Reprogramming (CEDEBIOR), Department of Biomedical Sciences, University of Sassari, Viale San Pietro 43/B, 07100 Sassari, Italy

**Keywords:** adipose stem cells, cell differentiation, gene expression, epigenetic, adipogenesis, conditioned media, inflammation, autophagy

## Abstract

Adipose-derived stem cells (ADSCs) came out from the regenerative medicine landscape for their ability to differentiate into several phenotypes, contributing to tissue regeneration both in vitro and in vivo. Dysregulation in stem cell recruitment and differentiation during adipogenesis is linked to a chronic low-grade inflammation and macrophage infiltration inside the adipose tissue, insulin resistance, cardiovascular disease and obesity. In the present paper we aimed to evaluate the role of metformin and vitamin D, alone or in combination, in modulating inflammation and autophagy in ADSCs during adipogenic commitment. ADSCs were cultured for 21 days in the presence of a specific adipogenic differentiation medium, together with metformin, or vitamin D, or both. We then analyzed the expression of FoxO1 and Heat Shock Proteins (HSP) and the secretion of proinflammatory cytokines IL-6 and TNF-α by ELISA. Autophagy was also assessed by specific Western blot analysis of ATG12, LC3B I, and LC3B II expression. Our results showed the ability of the conditioned media to modulate adipogenic differentiation, finely tuning the inflammatory response and autophagy. We observed a modulation in HSP mRNA levels, and a significant downregulation in cytokine secretion. Taken together, our findings suggest the possible application of these molecules in clinical practice to counteract uncontrolled lipogenesis and prevent obesity and obesity-related metabolic disorders.

## 1. Introduction

Adipose-derived stem cells (ADSCs) are largely involved in therapeutic applications and regenerative medicine, being linked to the maintenance of adipose tissue homeostasis and regeneration [1]. ADSCs are able to undergo adipogenic differentiation into mature adipocytes under specific stimuli from their microenvironment [2]. Adipogenesis is a well-regulated process, depending on several genes and epigenetic regulators [3,4]. White adipose tissue (WAT) is the most abundant adipose tissue in the human body, responsible for maintaining glucose homeostasis, energy balance, and hormone secretion [5]. Dysregulation in stem cell recruitment and differentiation leads to the secretion of pro-inflammatory cytokines, metabolic stress, insulin resistance, cardiovascular diseases, and obesity [6,7]. Uncontrolled activation of adipogenesis is linked to a chronic low-grade inflammation and macrophage infiltration inside the adipose tissue. Loss of adipocyte functionality, as it occurs in obesity, is accompanied by the secretion of proinflammatory cytokines, as tumor necrosis factor alpha (TNF-α) and interleukin 6 (IL-6) [5,8,9]. Moreover, adipocyte hypertrophy leads to a disequilibrium between lipogenesis and lipolysis, and alterations in the signal transduction process, damaging other organs and tissues [10,11]. Forkhead box-O1 (FoxO1) is a transcription factor exerting an essential role in controlling lipid metabolism and energy homeostasis, modulating adipocyte terminal differentiation [12,13]. FoxO1 activity has been also involved in tuning WAT/BAT differentiation, through a direct induction of autophagosome formation, which has been linked to the downregulation of UCP1 [13,14]. WAT browning is a complex process of mature white adipocyte transdifferentiation into “brown-like” or beige adipocytes [15], representing a valuable strategy to counteract obesity [16]. Beige adipocytes share some common features with brown adipose tissue (BAT), such as increased energy expenditure due to the greater number of mitochondria [17]. Additionally, WAT browning decreases inflammation by reducing the release of pro-inflammatory cytokines and regulating the expression of specific Heath Shock Proteins (HSP) [16,18,19]. HSP60 is the main mediator of adipose tissue inflammation. High concentrations of HSP60 in adipocytes contribute to lipid accumulation and the development of inflammatory processes [20,21]. Moreover, HSP70 seems to be involved in regulating WAT and BAT differentiation. In particular, HSP70 shows higher levels of expression in BAT, facilitating the thermogenic function of this tissue, while being downregulated in obesity [22,23]. Furthermore, HSP70 interacts with several transcription factors in order to modulate stem cell behavior and autophagy [24,25]. Autophagy is a crucial cell survival mechanism, controlling stem cell self-renewal and differentiation. Autophagy also supports adipogenesis, maintaining the balance between white and brown adipose tissue [26,27,28]. Increased autophagy occurs together with adipose tissue inflammation in WAT [29], while its suppression in BAT improves energy metabolism and insulin sensitivity by regulating mitochondrial turnover [30]. Several molecules are able to inhibit unproper differentiation and activation of ADSCs into white adipocytes, suggesting their potential application in preventing and managing obesity and obesity-related disorders [31,32]. We previously demonstrated that the combination of vitamin D and metformin can counteract the appearance of a white adipogenic phenotype, despite the presence of a specific adipogenic conditioned medium, by enhancing vitamin D metabolism, acting on CYP27B1 and CYP3A4 [33]. In the present study, we evaluated the effects of these two molecules, alone or in combination, in modulating stem cell behavior during adipogenic commitment, with particular attention to cytokine release and autophagosome formation, in the attempt to balance WAT/BAT differentiation and counteract uncontrolled lipogenesis. 

## 2. Results

### 2.1. Metformin and Vitamin D Inhibit ADSC Adipogenic Differentiation

**Figure 1:**
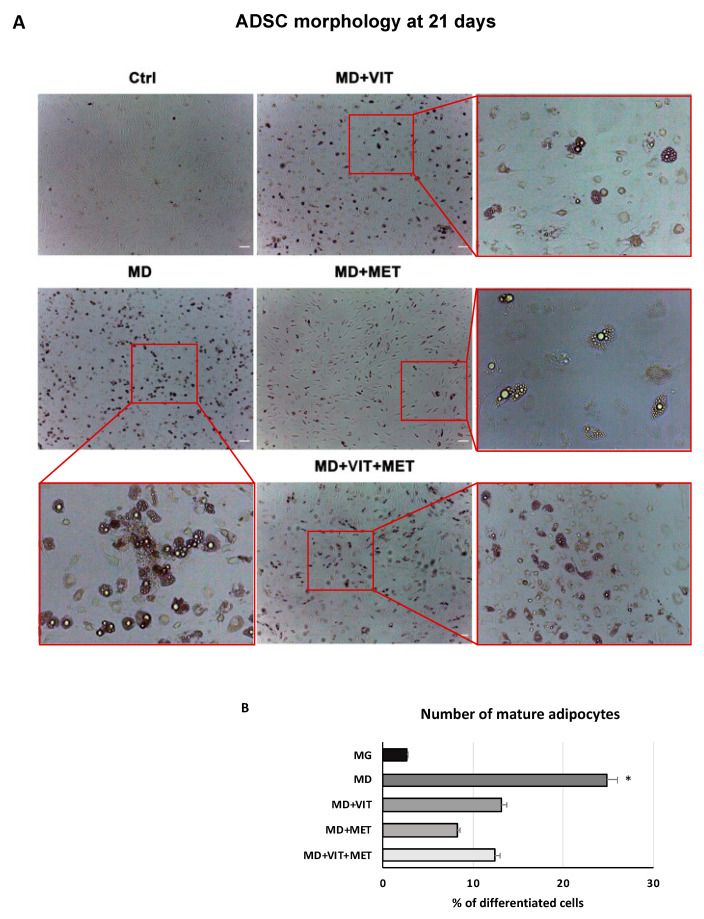
Optical microscope analysis of ADSC morphology during differentiation: (**A**) Figure shows morphological changes in cells treated with adipogenic differentiation medium (MD), or in MD plus vitamin D (MD+VIT), or in MD plus metformin (MD+MET), or in MD with both metformin and vitamin D (MD+VIT+MET), as compared to control untreated cells (Ctrl). ADSCs cultured in adipogenic medium alone acquired the appearance of mature adipocytes (MD). Scale bar = 100 µm. (**B**) The number of mature adipocytes was calculated using ImageJ. Data are expressed as mean ± SD referred to the control (* *p* ≤ 0.05).

ADSC morphology after 21 days of differentiation was evaluated by optical microscopy. Figure 1 shows significant changes in the morphology of ADSCs cultured in adipogenic medium (MD) containing vitamin D (MD+VIT), or metformin (MD+MET), or both (MD+VIT+MET), with a reduced number of adipocytes, as compared to untreated control cells (Ctrl). The same Figure shows that ADSCs cultured in MD alone exhibited a typical mature adipocyte morphology (Figure 1). Our results are further inferred by previous observation by our group [33], in which we demonstrated the ability of these two molecules to oppose adipogenic differentiation, albeit in the presence of a specific adipogenic conditioned medium. 

### 2.2. The Combination of Metformin and Vitamin D Inhibit the Release of Pro-Inflammatory Cytokines

**Figure 2:**
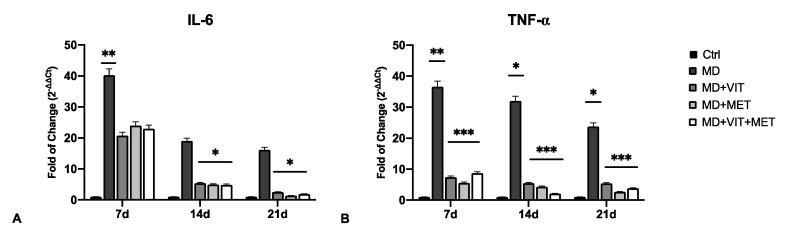
The expression of IL-6 (**A**) and TNF-α (**B**) was evaluated after 7, 14 and 21 days in ADSCs cultured in the presence of adipogenic differentiation medium (MD) (blue bars), or in MD plus vitamin D (MD+VIT) (yellow bars), or in MD plus metformin (MD+MET) (orange bars), or in MD with both metformin and vitamin D (MD+VIT+MET) (red bars), as compared to control untreated cells (grey bars). The mRNA levels for each gene were normalized to Glyceraldehyde-3-Phosphate-Dehydrogenase (GAPDH) and expressed as fold of change (2^−∆∆Ct^) of the mRNA levels observed in undifferentiated control ADSCs defined as 1 (mean ± SD; n = 6). Data are expressed as mean ± SD referred to the control (* *p* ≤ 0.05; ** *p* ≤ 0.01; *** *p* ≤ 0.001).

**Figure 3:**
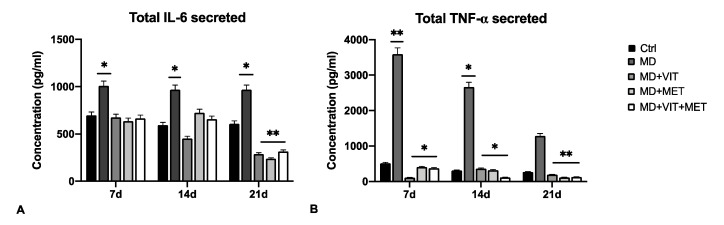
IL-6 and TNF-α quantification by ELISA: The concentration of IL-6 (**A**) and TNF-α (**B**) was measured after 7, 14, and 21 days in supernatants of ADSCs cultured in the presence of adipogenic differentiation medium (MD) (blue bars), or in MD plus vitamin D (MD+VIT) (yellow bars), or in MD plus metformin (MD+MET) (orange bars), or in MD with both metformin and vitamin D (MD+VIT+MET) (red bars), as compared to control untreated cells (grey bars). Data are expressed as mean ± SD referred to the control (mean ± SD; n = 6) (* *p* ≤ 0.05; ** *p* ≤ 0.01).

The expression of proinflammatory cytokines IL-6 and TNF-α was evaluated by qPCR (Figure 2) and ELISA (Figure 3) in ADSCs cultured in different conditioned media after 7, 14, and 21 days. The mRNA levels of IL-6 significantly decreased after 14 days of differentiation (Figure 2A), as compared to cells exposed to MD alone. TNF-α was also significantly downregulated even after 7 days of exposure when metformin, or vitamin D, or both, were added to MD (Figure 2B). These results were further confirmed by ELISA, showing significantly reduced concentrations of IL-6 and TNF-α in supernatants of cells exposed to metformin, or vitamin D, or both (Figure 3). The release of IL-6 was significantly reduced at the end of the differentiation period (21 days) (Figure 3A), while TNF-α showed a significant inhibition during all of the analyzed time points (Figure 3B), as compared to ADSCs exposed to MD alone. 

### 2.3. Exposure to Metformin Alone or Together with Vitamin D Modulates the Expression of HSPs and Autophagy 

**Figure 4:**
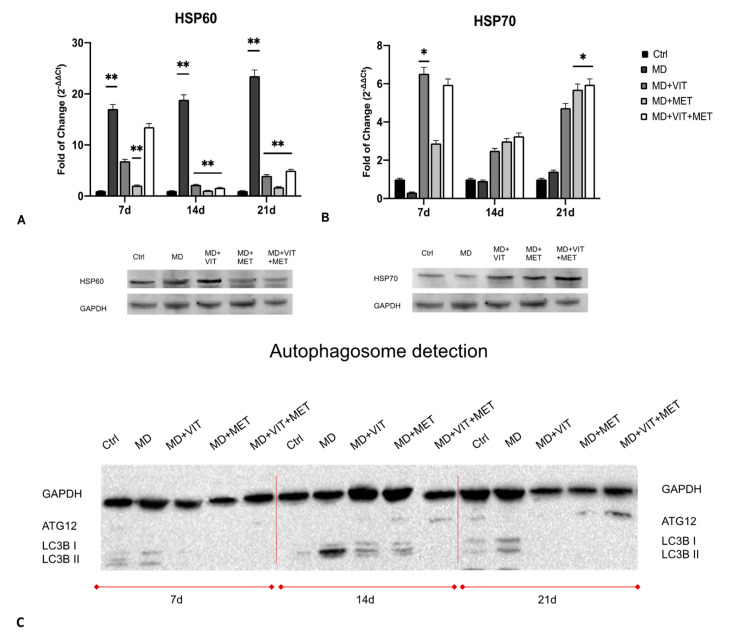
Analysis of heat shock proteins: The expression of HSP60 (**A**) and HSP70 (**B**) was evaluated after 7, 14, and 21 days in ADSCs cultured in the presence of adipogenic differentiation medium (MD) (blue bars), or in MD plus vitamin D (MD+VIT) (yellow bars), or in MD plus metformin (MD+MET) (orange bars), or in MD with both metformin and vitamin D (MD+VIT+MET) (red bars), as compared to control untreated cells (grey bars). The mRNA levels for each gene were normalized to Glyceraldehyde-3-Phosphate-Dehydrogenase (GAPDH) and expressed as fold of change (2^−∆∆Ct^) of the mRNA levels observed in undifferentiated control ADSCs defined as 1 (mean ±SD; n = 6). Data are expressed as mean ± SD referred to the control (* *p* ≤ 0.05; ** *p* ≤ 0.01). (**C**) Analysis of autophagosome formation. The protein levels were analyzed on ADSCs cultured in the presence of adipogenic differentiation medium (MD), or in MD plus vitamin D (MD+VIT), or in MD plus metformin (MD+MET), or in MD with both metformin and vitamin D (MD+VIT+MET), as compared to control untreated cells (Ctrl) after 7, 14, and 21 days by Western blot, using Autophagosome Marker Antibody Sampler Kit. The sizes of the bands were determined using pre-stained marker proteins. The data presented are representative of different independent experiments.

Figure 4 shows the levels of expression of HSP60 and HSP70 (panels A and B, respectively) in ADSCs cultured in the presence of different conditioned media. HSP60 expression was significantly increased in cells exposed to differentiation medium alone (MD), while in the presence of the other conditioned media (MD+VIT; MD+MET; MD+VIT+MET) their expression was similar to what was observed in control untreated cells (Figure 4A). An opposite trend was observed for HSP70, whose expression was significantly increased in ADSCs cultured in the presence of metformin (MD+MET), or both metformin and vitamin D (MD+VIT+MET) (Figure 4B), as compared to both control untreated cells and ADSCs cultured in the presence of MD alone. 

Western blotting analysis of autophagosome formation (Figure 4C) showed the activation of autophagy in ADSCs cultured in the presence of the adipogenic differentiation medium alone (MD) for all the analyzed time points, with a marked expression of LC3I and LC3II proteins, as compared to control untreated cells (Ctrl). The exposure to vitamin D (MD+VIT), or metformin (MD+MET), or both (MD+VIT+MET) inhibited autophagosome formation at the end of the 21 days of differentiation. The ATG12 protein, an Ubiquitin-like protein involved in autophagy vesicles formation and controlling MSC behavior [34] showed a marked expression in cells exposed to both vitamin D and metformin (MD+VIT+MET) at 14 and 21 days, as compared to both control untreated cells and cells exposed to MD alone. 

### 2.4. Exposure to Metformin Alone or Together with Vitamin D Modulates the Expression of FoxO1

Immunohistochemical analysis showed that in the presence of metformin alone (MD+MET) or together with vitamin D (MD+VIT+MET), the expression of FoxO1 was downregulated, as compared to cells cultured in the presence of adipogenic differentiation medium alone (MD). On the other hand, FoxO1 expression was increased in cells exposed to the differentiation medium in the presence of vitamin D alone (MD+VIT). 

## 3. Discussion

**Figure 5:**
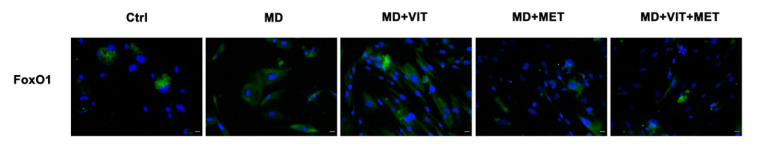
Immunohistochemistry analysis of FoxO1 after 21 days of differentiation: Immunohistochemical analysis of the expression of FoxO1 was performed in ADSCs cultured in the presence of adipogenic differentiation medium (MD), or in MD plus vitamin D (MD+VIT), or in MD plus metformin (MD+MET), or in MD with both metformin and vitamin D (MD+VIT+MET), as compared to control untreated cells (Ctrl). The figures are representative of two different independent experiments with three technical replicates. For each differentiation marker, fields with the highest yield of positively stained cells are shown. Nuclei are labelled with 4,6-diamidino-2-phenylindole (DAPI, blue). Scale bars: 40 µm.

Adipose-derived stem cells have a great plasticity, being able to differentiate into several phenotypes taking part in tissue regeneration both in vivo and in vitro [35]. In physiological conditions, ADSCs are located inside of the stromal vascular fraction (SVF) of the adipose tissue and are pre-committed cells, supporting adipogenesis and fatty acid accumulation [36,37]. Several transcriptional programs are activated or inhibited during differentiation, involving stemness genes, specific tissue markers and epigenetic modulators [38]. In particular, during adipogenesis, ADSCs exhibit high levels of the main adipogenic-related markers, peroxisome proliferator-activated receptor γ (PPAR-γ), fatty acid binding protein (FABP) 4, also known as aP2, lipoprotein lipase (LPL) and acyl-CoA thioesterase 2 (ACOT2) [31]. We have previously demonstrated that the combination of natural molecules, for example, melatonin and vitamin D together with the adipogenic conditioned medium, can counteract the appearance of an adipogenic phenotype in ADSCs, instead stimulating osteogenic differentiation [32]. Moreover, metformin, widely known in the treatment of obesity-related diabetes, promotes stem cell differentiation [39]. In addition, metformin reduced the levels of TNF-α in obese mice, down-regulating NF-kB translocation into macrophages [40]. We recently described the effect of metformin, alone or in combination with vitamin D, in controlling ADSC adipogenic differentiation, acting on vitamin D metabolism through epigenetic modification and miRNAs [33]. Within this context, in the present study, we aimed to evaluate the ability of the two molecules to modulate inflammation and autophagy, both closely related to obesity and unproper activation of adipocytes. Uncontrolled accumulation of adipose tissue occurring in obesity leads to the onset of a whole series of metabolic syndrome and pathological conditions [9]. In addition, the establishment of a state of chronic low-grade inflammation induces the infiltration of bone marrow-derived immune cells, negatively impacting organ function [41]. Our results showed a reduced number of mature adipocytes when ADSCs were cultured in the presence of vitamin D, or metformin, or both (Figure 1), despite the presence of the adipogenic conditioned media, as compared to cells exposed to MD alone, showing the typical morphology of mature adipocytes. Moreover, metformin and vitamin D are able to modulate the inflammatory response, acting directly on the secretion of the proinflammatory cytokines IL-6 and TNF-α (Figure 2). Actually, the activity of the two molecules resulted in a significant downregulation in the expression levels of these cytokines from the first days of differentiation, as compared to cells cultured in the presence of the MD alone. These results were further inferred by the ELISA assay, which revealed a significant inhibition of cytokine release in cells exposed to metformin or vitamin D or both (Figure 3), as compared to both untreated control cells and ADSCs cultured in the presence of MD alone. Moreover, heath shock proteins have a central role in regulating adipose tissue inflammation and homeostasis [42]. In particular, high expression of HSP60 induces the release of IL-6 and TNF-α by adipocytes, contributing to the onset of insulin resistance [21]. Furthermore, HSP60 concentrations are strictly related to triglyceride accumulation or decrease after surgery-induced weight loss [43]. Here, we provide evidence that ADSC exposure to metformin and vitamin D during adipogenic commitment is able to significantly downregulate HSP60 levels of expression (Figure 4A), akin to the observed decrease in cytokine production. An opposite trend was observed for HSP70, whose expression is upregulated in cells exposed to both metformin and vitamin D (Figure 4B). Increased levels in HSP70 in BAT might regulate T cell-mediated inflammation, protecting mitochondria and cells from apoptosis [44,45]. Moreover, HSP70 is involved in autophagy regulation in several physiological processes [46]. Autophagy is required for lipid storage during white adipocyte differentiation [47]. Hyperactivation of autophagy was observed in adipocyte hypertrophy, and obese and diabetic patients [48]. WAT to beige or BAT transdifferentiation has been recognized as a potential therapeutic target for obesity and related metabolic disease prevention and management [49]. Metformin might increase BAT thermogenic markers and mitochondrial biogenesis, promoting brown adipocyte proliferation and differentiation [50]. Inhibition of autophagy leads to increased levels of uncoupling protein 1 (UCP1), which regulates the browning of WAT and beige adipocytes [26,51]. The downregulation of LC3I/LC3II, assessed by Western blot (Figure 4C), seems to indicate that metformin and vitamin D inhibit autophagosome formation in ADSCs during adipogenic commitment. This event, related to the modulation of HSP60 and 70 (Figure 4C), highlights a possible role of metformin and vitamin D in counteracting WAT formation while inducing BAT differentiation. At the same time, ADSCs exposed to MD+VIT+MET showed a higher expression of ATG12, taking part in regulating mitochondrial biogenesis and cellular energy metabolism [52]. This temporary shutdown in autophagosome formation, while keeping ATG12 expression, could represent the molecular switch in the transition from white to brown adipose tissue. These events appear to be directly mediated by FoxO1, whose expression changes significantly depending on the presence of the different molecules in the conditioned medium. Figure 5 shows that FoxO1 is upregulated during adipogenic differentiation (MD), confirming previous findings by other authors [53]. Moreover, it has been demonstrated that vitamin D can promote bone formation and glucose homeostasis by activating FoxO1 [54]. On the other hand, inhibition of FoxO1 suppresses autophagy, increasing UCP1 expression [55]. Our results showed that treatment with metformin is able to inhibit FoxO1 expression, closely controlling adipocyte differentiation, even in the presence of vitamin D activation. 

## 4. Materials and Methods

### 4.1. Cell Isolation and Culturing Conditions 

ADSCs were isolated from subcutaneous adipose tissue of men and women (n = 6, age = 45 ± 15 years, BMI: 22 ± 3 kg/m^2^) after written informed consent. The study was approved by the Review Board of the Human Studies Ethics Committee of Sassari (n° ETIC 240I/CE 26 July 2016, Ethical committee, ASL Sassari). Immediately after harvesting, samples of adipose tissue were washed in PBS (Euroclone, Milan, Italy), minced into small fragments and digested by type I Collagenase solution for 1 h at 37 °C (Gibco Life Technologies, Grand Island, NY, USA) as previously described [28]. Cells were then centrifuged and resuspended in a basic growing medium consisting of Dulbecco’s modified Eagle’s medium (DMEM) (Life Technologies Grand Island, NY, USA) supplemented with 20% fetal bovine serum (FBS) (Life Technologies, Grand Island, NY, USA), 200 mM L-glutamine (Euroclone, Milan, Italy), and 200 U/mL penicillin 0.1 mg/mL streptomycin (Euroclone, Milan, Italy). The growing medium was changed every 3 days and, when cells reached the confluence, were trypsinized and immunomagnetically separated for flow cytometry characterization, as previously described [28]. Cells at passage 5 used as untreated controls were maintained in basic growing medium (Ctrl). A group of cells was cultured in a specific adipogenic differentiation medium (MD) (StemPro Adipocyte Differentiation Medium, Gibco Life Technologies, Grand Island, NY, USA) and used as positive control for adipogenic differentiation. Finally, a group of cells was cultured in MD in the presence of 10^−6^ M vitamin D (Sigma–Aldrich Chemie GmbH, Munich, Germany) (MD+VIT) or 5 mM metformin (Sigma–Aldrich Chemie GmbH, Munich, Germany) (MD+MET) or both (MD+VIT+MET). All experiments were performed twice (in three technical replicates).

### 4.2. Gene Expression Analysis

Gene expression analysis was performed after 7, 14, and 21 days in cells cultured under the above-described conditions. Total RNA was extracted using the ChargeSwitch kit (Thermo Fisher Scientific, Grand Island, NY, USA) according to the manufacturer’s instructions, and quantified by the NanoDrop™ One/OneC Microvolume UV-Vis spectrophotometer (Thermo Fisher Scientific, Grand Island, NY, USA). Approximately 1 µg of total RNA was reverse transcribed using the High-Capacity cDNA Reverse Transcription Kit (Thermo Fisher Scientific, Grand Island, NY, USA). Real-time quantitative PCR was performed by Luna^®^ Universal qPCR Master Mix (New England Biolabs, Ipswich, MA, USA) in triplicate using a CFX Thermal Cycler (Bio-Rad, Hercules, CA, USA). Amplification cycling was carried out at 95 °C for 60 s, then cycled at 95 °C for 15 s and 60 °C for 30 s, for a total of 40–45 cycles. Target Ct values of each sample were normalized to hGAPDH, which was considered as a reference gene. The relative values of the genes of interest, Interleukin 6 (IL-6), tumor necrosis factor alpha (TNF-α), Heat Shock Protein 60 (HSP60), and Heat Shock Protein 70 (HSP70) were expressed as fold of change (2^−∆∆Ct^) of mRNA levels observed in undifferentiated ADSCs, used as untreated control cells. All primers used (Thermo Fisher Scientific, Grand Island, NY, USA), are reported in Table 1.

### 4.3. ELISA Assay

The concentrations of IL-6 and TNF-α were determined using streptavidin-HRP conjugated systems Human IL-6 Mini TMB ELISA Development kit (PeproTech EC, Ltd., London, UK) and Human TNF-α Mini TMB ELISA Development kit (PeproTech EC, Ltd., London, UK), respectively. Cell culture supernatants were collected after 7, 14, and 21 days from ADSCs cultured under the above-described conditions. Exactly 100 μL of each sample was incubated in a pre-treated plate for 2 h at RT. After three washing steps in PBS, detection antibody was added in each well for 2 h at RT and then removed and replaced by streptavidin-HRP for 30 min at RT. Antibody was then washed three times and liquid substrate incubated at RT for 20 min. Color development was analyzed at 450 nm using a plate reader (Akribis Scientific, Common Farm, Frog Ln, Knutsford, UK). Standard curves were prepared according to manufacturer’s instructions. Each sample was assayed in duplicate, and values were expressed as the mean ± SD of 2 measures per sample.

### 4.4. Autophagosome Detection Assays

For evaluation of autophagosome formation by Western blot, the Autophagosome Marker Antibody Sampler Kit (Cell Signaling Technology, Danvers, MA, USA) was used. Protein extraction was performed according to manufacturer’s instructions from ADSCs cultured in the above-described conditions after 7, 14, and 21 days. After lysis by adding 1X SDS sample buffer, samples were heated to 95–100 °C for 5 min and then loaded onto SDS-PAGE gel. Proteins were electrotransferred to a nitrocellulose membrane using iBlot^®^ Dry Blotting System (Thermo Fisher Scientific, Grand Island, NY, USA. The membrane was incubated in blocking buffer for 1 h at room temperature and then ON at 4 °C in primary antibodies ATG12, LC3 I and II. At the end of incubation time, the membrane was washed three times in TBS and incubated in with HRP-conjugated secondary antibody for 1 h at RT. Protein expression was assessed by SuperSignal Chemiluminescent HRP Substrates (Thermo Fisher Scientific, Grand Island, NY, USA). 

### 4.5. Immunostaining

At the end of 21 days of differentiation in the above described conditions, ADSCs were fixed in paraformaldehyde (Sigma–Aldrich Chemie GmbH, Germany) for 30 min at RT with 4% and permeabilized with 0.1% Triton X-100 (Thermo Fisher Scientific, Grand Island, NY, USA)-PBS. After three washings in PBS, cells were incubated in 3% bovine serum albumin (BSA)-0.1% Triton X-100 in PBS (Thermo Fisher Scientific, Grand Island, NY, USA) for 30 min. A FoxO1 (C29H4) rabbit primary antibody was incubated ON at 4 °C. At the end of incubation, cells were washed three times in PBS for 5 min and incubated with fluorescence-conjugated secondary antibodies (Life Technologies, USA) at 37 °C for 1 h in the dark. Nuclei were labeled with 1 µg/mL 4,6-diamidino-2-phenylindole (DAPI) (Thermo Fisher Scientific, Grand Island, NY, USA). Fluorescence was acquired with a confocal microscope (TCS SP5, Leica, Nussloch, Germany). 

### 4.6. Statistical Analysis

Statistical analysis was performed using GraphPad Prism 9.0 software (GraphPad, San Diego, CA, USA). For each treatment, two separated experiments with three technical replicates were performed. Two-way analysis-of-variance ANOVA tests with Tukey’s correction and the Wilcoxon signed-rank test were used, assuming a *p* value < 0.05 as statistically significant. We considered * *p* < 0.05, ** *p* < 0.01, *** *p* < 0.001.

## 5. Conclusions

Taken together, our findings suggest the possible application of metformin and vitamin D in controlling adipogenic differentiation, inhibiting WAT formation, and promoting BAT differentiation by temporary inactivating autophagosome formation and HSP modulation. The ability of these two molecules to control autophagy and inflammation could represent a novel target for the regulation of lipogenesis and treatment of obesity-related metabolic disorders.

## Figures and Tables

**Table 1:** Primer sequences.

Primer Name	Forward	Reverse
hGAPDH	GAGTCAACGGAATTTGGTCGT	GACAAGCTTCCCGTTCTCAG
IL-6	TCTCAACCCCAATAA	GCCGTCGAGGATGTA
TNF-α	CCTCAGACGCCACAT	GAGGGCTGATTAGAGAGA
HSP60	GGGCATCTGTAACTCTGTCTT	TAAAAGGAAAAGGTGACAAGG
HSP70	CACAGCGACGTAGCAGCTCT	ATGTCGGTGGTGGGCATAGA

## Data Availability

Not applicable.
